# Meditation experience predicts negative reinforcement learning and is associated with attenuated FRN amplitude

**DOI:** 10.3758/s13415-018-00665-0

**Published:** 2018-11-16

**Authors:** Paul Knytl, Bertram Opitz

**Affiliations:** School of Psychology, University of Surrey, Guildford, Surrey, GU2 7XH UK

**Keywords:** Feedback-learning bias, Feedback-related negativity, FRN, Reinforcement learning, Meditation, Dopamine, ACC, Striatum

## Abstract

Focused attention meditation (FAM) practices are cognitive control exercises where meditators learn to maintain focus and attention in the face of distracting stimuli. Previous studies have shown that FAM is both activating and causing plastic changes to the mesolimbic dopamine system and some of its target structures, particularly the anterior cingulate cortex (ACC) and striatum. Feedback-based learning also depends on these systems and is known to be modulated by tonic dopamine levels. Capitalizing on previous findings that FAM practices seem to cause dopamine release, the present study shows that FAM experience predicts learning from negative feedback on a probabilistic selection task. Furthermore, meditators exhibited attenuated feedback-related negativity (FRN) as compared with nonmeditators and this effect scales with meditation experience. Given that reinforcement learning and FRN are modulated by dopamine levels, a possible explanation for our findings is that FAM practice causes persistent increases in tonic dopamine levels which scale with amount of practice, thus altering feedback processing.

Since the turn of the century meditation has gone from relative obscurity in Western academia to explosive growth in interest and research. Only around 60 academic papers were published on the topic of mindfulness in 2003; by 2013 that number jumped to 600 (Shonin, Van Gordon, & Griffiths, 2013). The appeal is understandable; claimed benefits of meditation range from reduced stress (Kabat-Zinn, 2003) to improved immune function (Davidson et al., 2003), from improved attention and lower anxiety (Tang et al., 2007) to a novel treatment for depression (Teasdale et al., 2000), and has even been recommended as a possible way to manage symptoms of psychosis (Shonin, Van Gordon, & Griffiths, 2014).

While some researchers have been pressing forward exploring the possible applications of meditation, others have been trying to understand what exactly is occurring in the nervous system during and after meditation, what systems are involved, and what the long-term effects of practice might be. In part due to methodological issues, inconsistent operationalization, the relative lack of longitudinal studies, and the infancy of the field, the precise mechanisms and long- term effects of various meditation styles are still not entirely clear (Cahn & Polich, 2006; Hölzel et al., 2011; Tang, Hölzel, & Posner, 2015; Vago & Silbersweig, 2012).

There has been recognition that effective study of these practices requires precise operationalization of the concept. The term *meditation* refers to a diverse group of cognitive practices which share some similarities and some fundamental differences (Lutz, Slagter, Dunne, & Davidson, 2008). To the uninitiated, the matter is further confused by the casual use of relevant terms by both the public and academia. For example, the term *mindfulness* can refer to a type of meditation, a trait, and a state of mind (Vago & Silbersweig, 2012). To address this, a framework has been introduced which categorizes meditative practices into three groups based on their primary cognitive strategy: focused attention meditation (FAM), open-monitoring meditation (OMM), and loving-kindness meditation (LKM; Hölzel et al., 2011; Lutz et al., 2008; Vago & Silbersweig, 2012). Of particular interest in the present study is FAM. FAM is central to many meditative traditions, such as Buddhist samatha and vipassana meditation and their derivative secular mindfulness practices and clinical interventions such as mindfulness-based stress reduction (MBSR), mindfulness-based cognitive therapy (MBCT), and other practices such as transcendental meditation (Harvey, 2015; Lutz et al., 2008; Vago & Silbersweig, 2012). FAM is characterized by the establishment, monitoring, and maintenance of attention on a chosen sensory object, such as the sensation of breathing (Lutz et al., 2008).

What is striking about FAM is that the cognitive processes invoked during practice bear close resemblance to the processes that the brain’s mesencephalic dopamine system and its target areas are thought to perform. For instance, others have hypothesized that the continual establishment, monitoring, and reestablishment of attention on an object of meditation during FAM should elicit activity in those brain areas already associated with conflict monitoring and sustained attention, such as the dorsolateral prefrontal cortex (dlPFC) and the anterior cingulate cortex (ACC; Lutz et al., 2008). An early review of 12 neuroimaging studies of meditation found numerous brain areas active during meditation, such as the striatum, hippocampus, thalamus, along with the ACC and dlPFC (Cahn & Polich, 2006). As this review predates Lutz et al.’s (2008) operationalization, it does not directly specify what kind of meditation may be involved (FAM, OMM, LKM, etc.), even including some studies of Christian prayer. This inclusion of a wide range of practices involved in the reviewed studies may account for the diverse brain areas reportedly active in meditators. Studies looking only at practices having a clear FA component (e.g., Buddhist and secular mindfulness) consistently report brain areas involved in attention and conflict monitoring, including the dlPFC and particularly the ACC, to be reliably active in meditators (for a review, see Tang et al., 2015).

There is also evidence of morphological differences and changes in plasticity in FAM practitioners. A recent anatomical likelihood estimation (ALE) meta-analysis of 21 morphometric brain imaging studies revealed higher grey and white matter density in the ACCs of meditators compared with nonmeditators (Fox et al., 2014). Neuroplastic changes may also happen relatively quickly after beginning FAM training; one intervention with an FAM component resulted in higher connectivity between the ACC and the brain stem after only 11 hours of practice (Tang et al., 2010). These findings suggest that meditation not only activates areas vital to attention but can also quickly induce neuroplastic growth in these brain regions.

Neuroimaging studies have also revealed that the striatum, a core component of the dopamine system, is active during meditation. In one study, [^11^C]raclopride, a radio ligand that binds competitively with dopamine D2 receptors, had been used to measure participants’ dopamine tone in the striatum, first while they listened to speech with their eyes closed and then while they were actively meditating (Kjaer et al., 2002). Compared with the speech state, the meditation state was associated with a 65% increase in endogenous dopamine release. In another investigation, fMRI was used to carry out a case study of an experienced Buddhist meditator while he used various meditative techniques including FAM (Hagerty et al., 2013). The authors reported that this participant displayed increased activity in the ACC and in the striatum during the FAM phase. Both of these studies suggest that practicing FAM activates the dopaminergic system in the cortex and basal ganglia.

The dopaminergic system has been shown to play an important role in reward, learning, error correction, motor control, and attention (Schultz, 2002). More precisely, phasic (fast) bursts of dopamine act as a learning signal which serve to orient organisms to novel or unexpected stimuli and encodes the difference between the expected and actual outcome of an action, a reward prediction error (RPE), in the form of either a spike in dopamine if the event was rewarding, or a dip if the event was not (Schultz, 2002, 2013, 2016). A seminal study demonstrated that dysfunctional dopamine levels explain some perplexing cognitive differences between people with Parkinson’s disease (PD), where dopamine production is severely reduced, and age and IQ matched controls (Frank, Seeberger, & O’Reilly, 2004). While off their dopaminergic medication, patients suffering from PD performed worse than controls on a probabilistic selection task (PST)—but only when they received positive feedback. When they received negative feedback they outperformed controls. When placed on L-DOPA medication, a dopamine precursor which increases tonic dopamine levels, the pattern reversed with PD patients outperforming controls on positive feedback-learning but underperforming on negative feedback-learning (Frank et al., 2004). Since a person can be said to have a feedback-learning bias (FLB) if they learn more effectively from either positive or negative feedback, this study demonstrated that up or down regulating tonic dopamine levels respectively creates a positive or negative feedback-learning bias.

The interaction between phasic dopamine bursts and persistent tonic dopamine levels provides a possible reason for this phenomenon. FLB has been explained using a neuro-computational model whereby an up-regulated or down-regulated tonic dopamine signal respectively enhanced or inhibited sensitivity to a particular type of feedback, thus creating a FLB (Frank, 2005). In this model, depressed tonic dopamine levels, such as those found in PD patients off medication, effectively increased the amplitude of phasic dopamine bursts required to activate the “go” pathway in the basal ganglia. At the same time, a smaller magnitude of phasic dip is required to trigger the threshold that leads to negative feedback learning, resulting in a negative FLB. Increasing tonic dopamine seems to have the opposite effect, amplifying the effect of phasic bursts, inhibiting the “no-go” pathway, and thus leading to a positive feedback-learning bias (Frank, 2005).

This phasic dopaminergic activity is widely believed to be the cause of one component of the event-related potential (ERP) known as feedback-related negativity (FRN). The FRN is typically calculated as the difference between negative-feedback and positive-feedback trial waveforms and is characterized by a frontocentral scalp distribution which peaks about 250 ms after feedback presentation (San Martín, 2012; Walsh & Anderson, 2012). The highly influential reinforcement learning (RL) theory of the FRN holds that phasic dips in dopaminergic activity (negative RPEs) disinhibit the ACC, which in turn generates the FRN (Holroyd & Coles, 2002). Hierarchical reinforcement learning (HRL) builds on the success of RL theory by breaking complex tasks into simpler sets of subroutines. This helps to manage the computational demand of real-world learning and decision-making scenarios, ensuring they are still tractable using the RL algorithm (Botvinick, 2012). The RL theory of the FRN has also been updated to leverage the insights gained from HRL (Holroyd & Yeung, 2012). This account elaborates on the role of the ACC, proposing that it is responsible for learning the values of, and maintaining engagement in, HRL subroutines. It draws on a number of more recent studies to argue that the ERN/FRN are in fact a function of an underlying component, sensitive to reward, termed the reward positivity (RewP), which reflects a positive, as opposed to negative, RPE. Although there has been some debate over whether the FRN reflects an underlying RPE, surprise, or response conflict, what remains unchanged and what is of paramount importance in the present study, is the abundance of evidence which supports the theory that the FRN is generated when the ACC responds to phasic dopaminergic inputs from the midbrain and basal ganglia (San Martín, 2012; Walsh & Anderson, 2012)

Given the tonic–phasic interaction in the dopamine system, it is not surprising that the FRN has also been linked to tonic dopamine levels. In one study, participants who displayed a positive FLB on the PST also presented a lower amplitude FRN than participants who were negatively biased (Frank, Woroch, & Curran, 2005). Other researchers have shown that people who are MET homozygous for the val158met COMT polymorphism, which elevates tonic dopamine levels, also present an attenuated FRN (Marco-Pallarés et al., 2009), and that dopamine agonists likewise reduce FRN amplitude (Santesso et al., 2009). These studies suggest that higher tonic dopamine levels result in not only a more positive feedback-learning bias but also a reduction in FRN amplitude.

Due to the overlapping systems involved in reinforcement learning and FAM, we hypothesized that FAM might induce a positive FLB and attenuated FRN by upregulating tonic dopamine levels. To investigate whether, compared with nonmeditators, the amount of FAM experience a person has would predict a more positive FLB and attenuated FRN, we carried out a study using a version of the probabilistic selection task (PST) employed by Frank et al. (2005). In this task participants were required to learn to choose the more rewarding of two symbols via trial and error. Positive and negative feedback was provided on a probabilistic basis, with some symbols being on average more rewarding than others. Therefore, when choosing a more rewarding symbol, participants were more likely to receive positive feedback than they would from a less rewarding one. We measured FLB and recorded their FRN while they completed this task. We reasoned that if FAM is causing long-term increases to tonic dopamine levels, meditators should exhibit characteristics of positively biased feedback learners on the PST as well as have an attenuated FRN. We predicted that, if this effect is caused by FAM practice, both the FLB and the FRN effects should scale with FAM experience.

## Method

### Participants

Thirty-five people were recruited from the local student population, Buddhist groups, and the general public. In keeping with the FAM/OMM/LKM framework, the 23 meditators among our participants consisted of Buddhist (*N* = 9), secular (*N* = 12), and Qi Gong (*N* = 2) FAM practitioners. Since the measures we used are sensitive to tonic dopamine levels, volunteers were prescreened using a self-report questionnaire for current episodes of major depression or anxiety, and monoamine oxidase inhibitors or recreational drug use.

The participants were grouped into a nonmeditator group (*N* = 12, mean age = 34.6 years, *SD* = 14.8 years, range: 22–60, six female), a novice meditator group (*N* = 12, mean age = 38.6 years, *SD* = 14.3 years, range: 18–59, 10 female) and an experienced meditator group (*N* = 11, mean age = 38.2 years, *SD* = 13.7 years, range: 23–56, six female) based on a median split^1^ on their self-reported meditation experience. We chose to use years of experience as our grouping variable in keeping with other studies (Kang et al., 2013). Participants were considered experienced if they meditated for more than 30 minutes a week for at least 4 years; this criterion reflects a median split of our data based on years of experience with the added proviso that meditators practiced on a weekly basis for a reasonable amount of time. Mean age was not significantly different between groups *F*(2, 32) < 1. A self-report prescreening questionnaire revealed that, except for one ambidextrous participant, all were right-handed. Everyone was a volunteer, and no compensation was provided beyond reimbursement of travel costs.

### Apparatus

Participants sat in a dimly lit, electrically shielded, sound attenuated booth. On a desk in front of them was a 17-in. LCD monitor used to display stimuli at a screen resolution of 1280 × 1024 at 60 Hz. The screen was approximately 1 meter from the subject’s face at eye level. Responses were recorded via keyboard. Besides the EEG amplifier and screen, all EMR sources were located outside the booth, and DC lighting was used.

### Design and procedure

This experiment used a between-subjects design examining the effect of three levels of FAM experience (control, novice, experienced) on two feedback learning measures: feedback-learning bias (FLB) and FRN amplitude (FRN).

We employed a version of the probabilistic selection task (PST) procedure used by Frank et al. (2005). During the EEG recording, participants were shown pairs of stimuli consisting of Japanese hiragana characters. Following the procedure from cited above, after a short practice block, stimuli were presented in three blocks each containing a training and test phase. Each block used one of three stimuli sets whose presentation order was randomized across participants.

In the training phase, participants were instructed to learn via trial-and-error to select the hiragana symbol most likely to provide positive feedback from each of three pairs (represented here using roman letters: *AB, CD, EF*). Pairs were presented in random order, and the side that the superior symbol appeared on was pseudorandomized such that symbols appeared equally often on either side of the screen. If no selection was made within 1,000 ms, the message “no response detected” was displayed. Participants were provided with positive or negative feedback to guide their learning; however, as summarized in Table 1, this feedback was probabilistic. For example, choosing *A* in the *AB* pair would result in positive feedback (+10 points, green colour, Arial, size matched to the hiragana characters) 80% of the time, whereas choosing *B* would result in positive feedback 20% of the time. In order to maximize their score in each training block, participants should have learned to choose *A* over *B*, *C* over *D*, and *E* over *F*. Note that this could be accomplished by either learning to select the winners (*A, C, E*), avoid the losers (*B, D, F*), or both. We enforced the same training criterion as Frank et al. (2005); after 60 trials, performance levels were checked (65% *A* in the *AB* pair, 60% *C* in *CD*, and 50% *E* in *EF*), and if the criterion was met, participants were released to the test phase. If the criterion was not met, participants would continue up to a maximum of 120 trials.

**Table 1:** Summary of the probability a participant would receive positive feedback when selecting a given character

Character	Probability of receiving positive feedback
A	0.80
B	0.20
C	0.70
D	0.30
E	0.60
F	0.40

During the test phase, participants were told to use their gut feeling to choose the superior symbol from the pairs presented, and this time no feedback would be provided. During this phase novel combinations (e.g., *AC* or *BD*) were presented to participants. This was done in order to assess which feedback type was most effective in teaching them and how accurate their learning was. Since *A* most reliably provided positive feedback and *B* most reliably provided negative feedback, participants should have learned to choose *A* whenever it was presented in a novel pair while avoiding *B* whenever it was presented in a novel pair. Our paradigm differed from the procedure in Frank et al. (2005) in two ways. First, we used points (+10 or −10 points for positive or negative feedback, respectively) instead of smiley faces, and *X* icons as the feedback graphics, as preexperimental paradigm testing revealed higher task engagement when a points system was used. Second, the number of pairings in the test phase containing *A* or *B* were increased by one, while all other pairings were reduced by one. This increased reliability without increasing task duration.

After participants had finished the task, they were provided with facilities to clean themselves up. They were then debriefed regarding the full nature and purpose of the experiment.

### Data recording and analysis

#### Behavioural data

The PST was created and run using the E-Prime 2.0 stimulus presentation suite. Responses from the training and test phase were recorded via keyboard. The mean accuracy scores across all pairs and blocks involving *A* and *B* were calculated. The FLB score is the mean difference, as a percentage, between correct Choose *A* and Avoid *B* responses. The *AB* trial was excluded from this calculation since the preferred strategy (Choose *A* or Avoid *B*) used to answer it cannot be dissociated.

#### Electrophysiological data

During the behavioural task, a 32-channel continuous EEG recording of each participant was made using Ag/AgCl electrodes. Electrodes were affixed to an appropriately sized EEG cap (EASYCAP GmbH) worn by each participant. The extended 10–20 electrode layout (Klem, Otto, Jasper, & Elger, 1999) was employed. Signals from two additional electrodes placed on the participant’s left and right mastoid were recorded. All electrodes recorded relative to a common average reference. Horizontal and vertical electrooculograms were recorded with electrodes located above and below the participant’s left eye, and on the outer canthi of both eyes. Interelectrode impedances were kept below 10 kΩ. All channels were amplified with a band pass from DC to 70 Hz and A/D converted with 16-bit resolution at a rate of 500 Hz.

ERPs were calculated using the off-line data processing suite included in the BrainAnalyzer2 program (Brain Products GmbH, Gilching, Germany). Data preprocessing consisted of a digital band-pass filter from 0.3 Hz to 30 Hz (−3 dB cut-off) to eliminate low-frequency signal drifts and high-frequency artefacts. Eye-movement artefacts were eliminated using an automated independent components approach as implemented in BrainAnalyzer2. Automatic artefact rejection (gradient criterion: voltage variation of more than 75 μV in two subsequent time points, amplitude criterion: any voltage exceeding ± 100 μV and low activity criterion: 0.5 μV/50 ms) was applied to all channels to mark segments contaminated by additional artefacts. These recording epochs were excluded from further analysis. Recordings were segmented with epochs ranging from −100 to +800 ms with respect to the onset of the feedback. Artefact-free segments from the training phases were averaged separately for each participant to form 4 ERPs by feedback type and valence (true positive, true negative, false positive, false negative). The 100-ms prior to feedback onset served as the baseline. These averages were digitally re-referenced to average mastoid activity.

We created the FRN difference wave by subtracting the true positive ERP from true negative ERP for each participant at the FCz electrode (e.g., Pfabigan, Alexopoulos, Bauer, & Sailer, 2011). We excluded false feedback trials from the analysis as recent work by Ernst and Steinhauser (2018) has shown that the FRN is attenuated in unreliable (i.e., false) feedback trials. As participants were informed in the present study that stimuli behaviour would be reliable most—but not all—of the time, we improved signal-to-noise ratio by excluding false feedback trials. The proportion of true feedback trials was constant at 70% across all participants.

Since persons exhibiting a positive bias on behavioural measures may have delayed FRNs (Frank et al., 2005), automatic peak detection was employed to pick the most negative value of the difference wave within an epoch ranging between 220 and 320 ms postfeedback. This epoch was selected as it should encapsulate peak FRN activity, approximately 265 ms poststimulus (Gehring & Willoughby, 2002; San Martín, 2012; Walsh & Anderson, 2012). The mean amplitude in a window ±50 ms of this peak was exported for analysis. To assess whether any change in the FRN across groups was driven by the ERP response to positive or negative feedback, the mean amplitude of the same time window around the FRN peak was also analysed for each condition specific ERP separately.

## Results

### Behavioural data

Average training-phase performance across all blocks and groups was 64%, indicating learning occurred. A 3 × 3 (Block × Group) Greenhouse–Geisser corrected (*ɛ* = .75) mixed-design ANOVA was conducted revealing no significant effect of block, *F*(1.50, 47.81) = 0.91, *p* = .38, group, *F*(2, 32) = 0.69, *p* = .51, or any interaction *F*(2.99, 47.81) = 0.56, *p* = .65, on training phase performance. There were no significant group differences for the ratio of true positive to true negative feedback received, *F*(2, 32) = 1.39, *p* = .26, or the ratio of total positive to total negative feedback received, *F*(2, 32) = 1.11, *p* = .34. Likewise, no group differences in the total number of trials performed *F*(2, 32) = 0.27, *p* = .76, were observed. In addition, overall test-phase performance did not differ between groups *F*(2, 32) = 0.05, *p* = .95.

The primary measure of interest from the behavioural data was feedback-learning bias (FLB). This quantity measures whether participants are biased towards learning Choose *A* or Avoid *B* behaviour. The value is computed by subtracting correct test-phase performance on Avoid *B* trials from Choose *A* trials. *AB* trial performance is excluded from this calculation, as it is not possible to disentangle which strategy (Choose *A*, or Avoid *B*) was used to learn the correct response. Thus, when FLB has a positive value, participants learned better from positive feedback; likewise, a negative value indicates negative feedback was more effective in training a participant.

The nonmeditators had the most negative FLB (*M* = −0.11, *SE* = 0.03). Meditators were positively biased, with the novice group (*M* = 0.00, *SE* = 0.04) being less positively biased than the experienced group (*M* = 0.05, *SE* = 0.06). An independent ANOVA was performed to determine the effect of meditation experience on FLB. As hypothesized, there was an effect of meditation experience on FLB, *F*(2, 32) = 3.41, *p* = .045, ƞ_p_^2^ = .18. Planned contrasts revealed that meditators were significantly more positively biased than were nonmeditators, *t*(32) = 2.51, *p* = .017, *r* = .41. However, no significant difference between the novice and experienced meditation groups, *t*(32) = 0.80, *p* = .43, *r* = .14, was found.

**Fig. 1:**
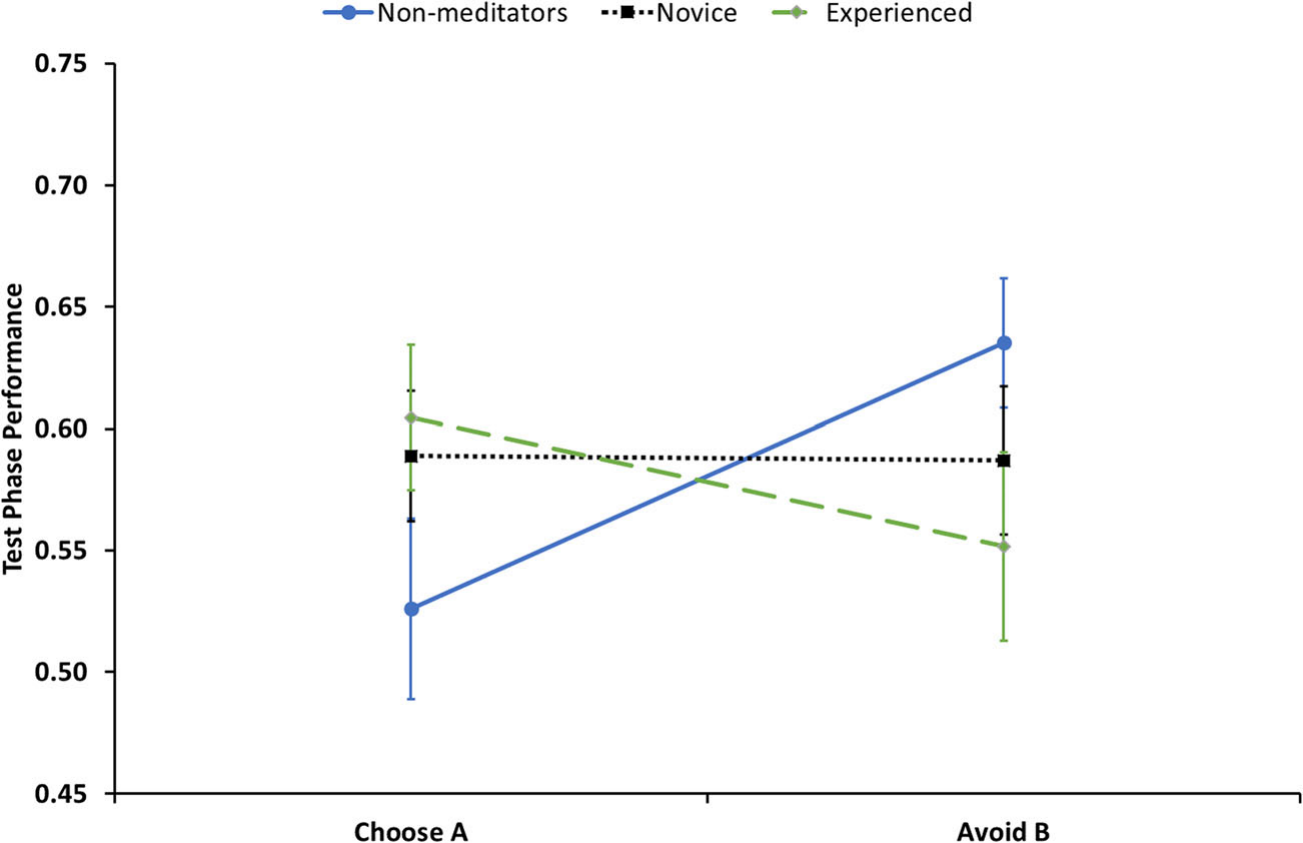
Choose *A* and Avoid *B* performance by group and feedback type. Error bars represent *SE*

We added participant age to the model to determine if this had an effect on FLB. The ANOVA revealed that while there was still a significant effect of meditation experience on FLB, *F*(2, 31) = 3.42, *p* = .045, ƞ_p_^2^ = .19, there was no significant effect of age, *F*(1, 31) = 0.22, *p* = .64, ƞ_p_^2^ = .007.

**Fig. 2:**
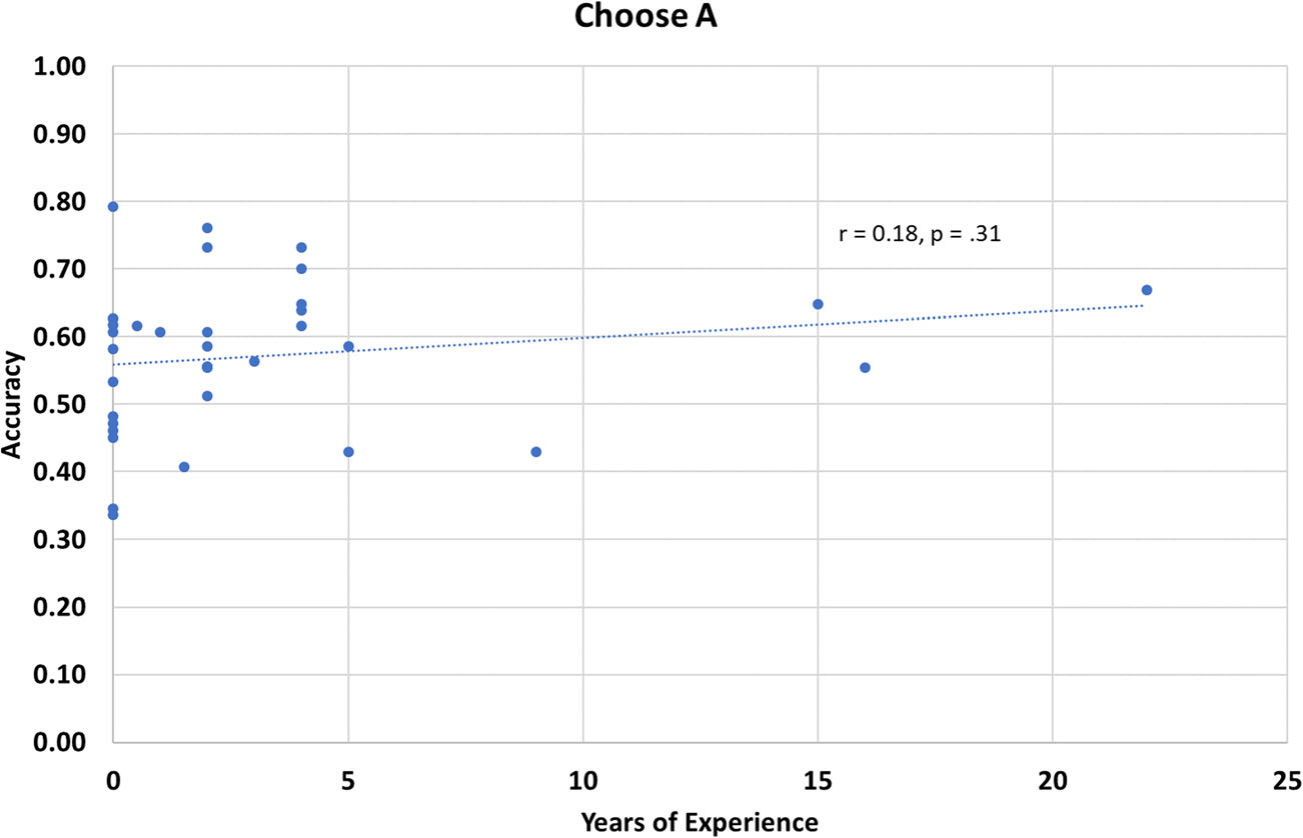
Choose *A* accuracy as a function of years of meditation experience.

**Fig. 3:**
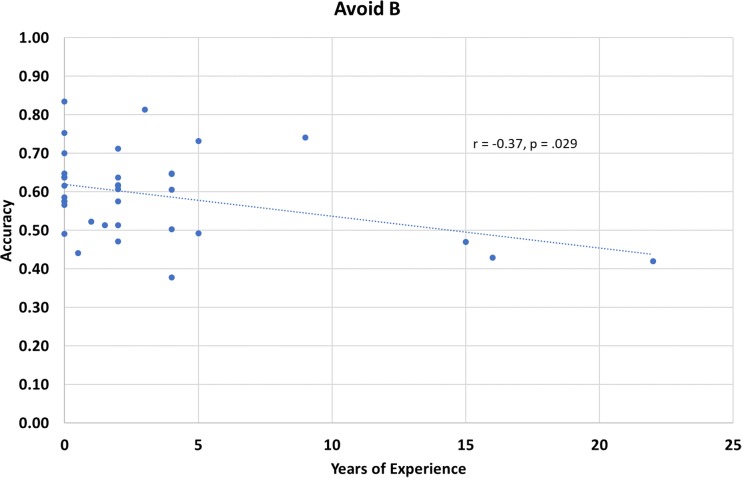
Avoid *B* accuracy as a function of years of meditation experience

A post hoc analysis was carried out in order to determine whether this shift in feedback processing was driven by an increase or decrease in Choose *A* or Avoid *B* performance. Approach and avoid performance is summarized by group in Fig. 1. The control group had the lowest Choose *A* test performance (*M = 0*.53, *SE* = 0.04), followed by the novice group (*M =* 0.59, *SE* = 0.03), while the experienced meditators had the best performance (*M =* 0.60, *SE =* 0.03). In terms of Avoid *B* performance, the control group had the highest performance (*M =* 0.64, *SE* = 0.03), followed by the novice group (*M =* 0.59, *SE* = 0.03), while the experienced meditators performed worst (*M =* 0.55, *SE =* 0.04). A 2 × 3 (Approach/Avoid × Meditation Group) mixed-design ANOVA was performed to determine whether there was an interaction between response behaviour type and meditation experience on participant test performance. There was no main effect of behaviour type, *F*(1, 32) = 0.50, *p* = .49, or meditation experience, *F*(2, 32) = 0.05, *p* = .95; however, there was a significant interaction between behaviour type and meditation experience on test performance *F*(2, 32) = 3.41, *p* = .045, ƞ_p_^2^ = .18. Based on our hypothesis that meditation may cause these effects, we conducted a post hoc linear regression to examine whether meditation experience independently predicts Choose *A* and Avoid *B* performance. Mediation experience did not significantly predict Choose *A* performance, *F*(1, 33) = 1.06, *p* = .31, *R*^*2*^ = .03 (see Fig. 2). However, meditation experience did predict Avoid *B* performance, *F*(1, 33) = 5.23, *p* = .029, *R*^*2*^ = .14 (see Fig. 3), indicating that as meditation experience increases, participants do not learn as well from negative feedback trials.

Overall, these results indicate that feedback-learning bias becomes more positive with meditation experience and that this cannot be explained by age. However, despite an upward trend, the fact that novice and experienced meditators were not significantly different on FLB is not supportive of our hypothesis that there would be a significant increase of this effect with meditation experience. Post hoc analysis revealed a significant interaction between approach and avoid performance and meditation experience, but despite trend-level evidence suggestive of equal contribution of changes in both Choose *A* and Avoid *B* performance, our sample lacked the power to further explicate the interaction at the group level.

### EEG data

For each participant, the main EEG measure was the scalp voltage at FCz, averaged across a 100-ms window centred on the peak of the FRN difference wave between 220 and 320 ms postfeedback. The difference wave was created by subtracting the true positive-feedback-locked waveform from the true negative-feedback-locked waveform at FCz.

**Fig. 4:**
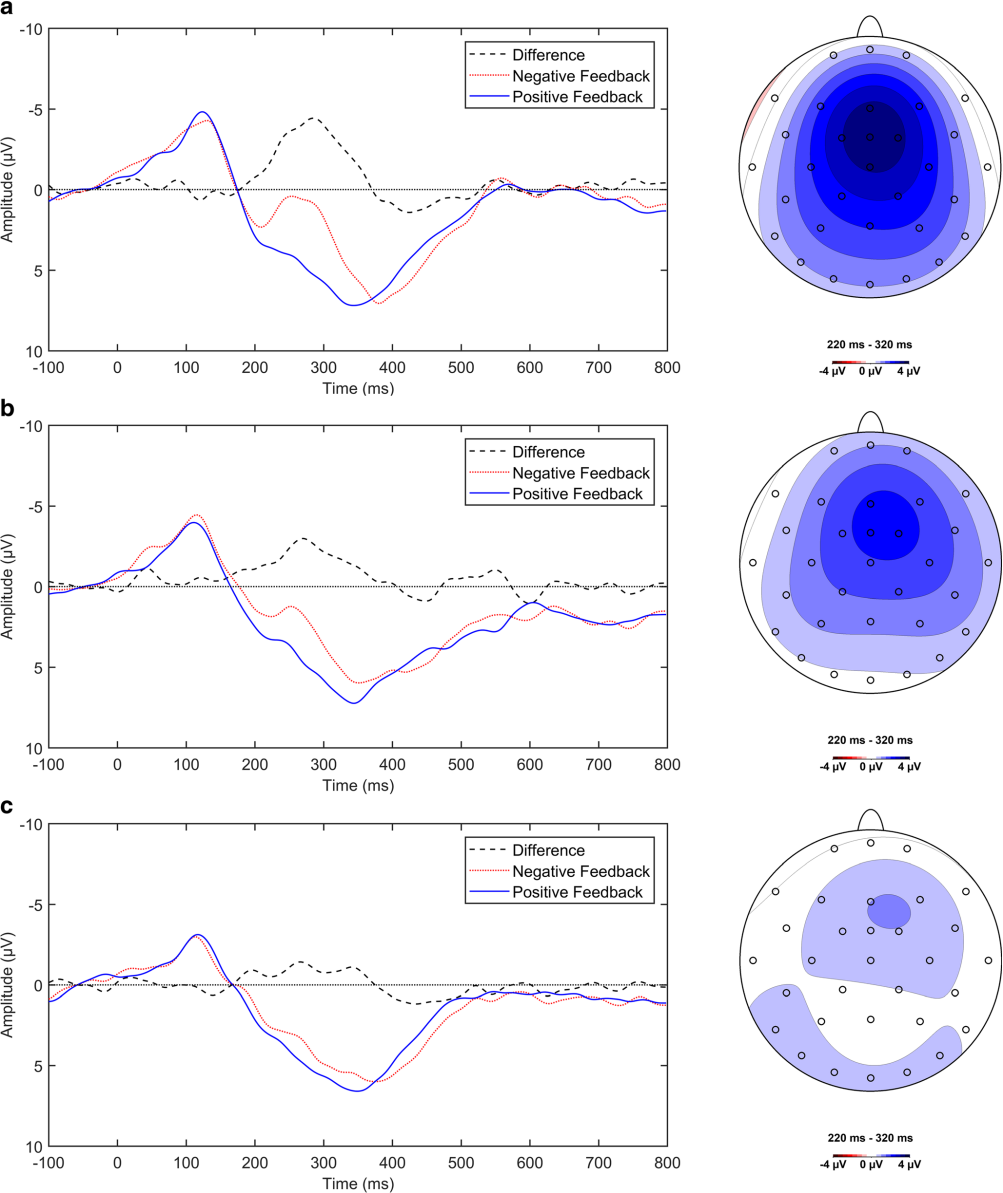
Feedback-locked FRN difference wave and condition specific ERPs at FCz elicited by true feedback trials for (**a**) the nonmeditation group, (**b**) the novice group, and (**c**) the experienced group. Following EEG convention, up is negative. Feedback stimulus occurs at zero ms. Negative-feedback-elicited waveform in blue, positive-feedback elicited waveform in red. Note the difference wave attenuates between 220 and 320 ms as meditation experience increases. Also shown, scalp distribution of the difference wave for each group. Peak activity has the frontomedial distribution and timing characteristic of the FRN. (Colour figure online)

**Fig. 5:**
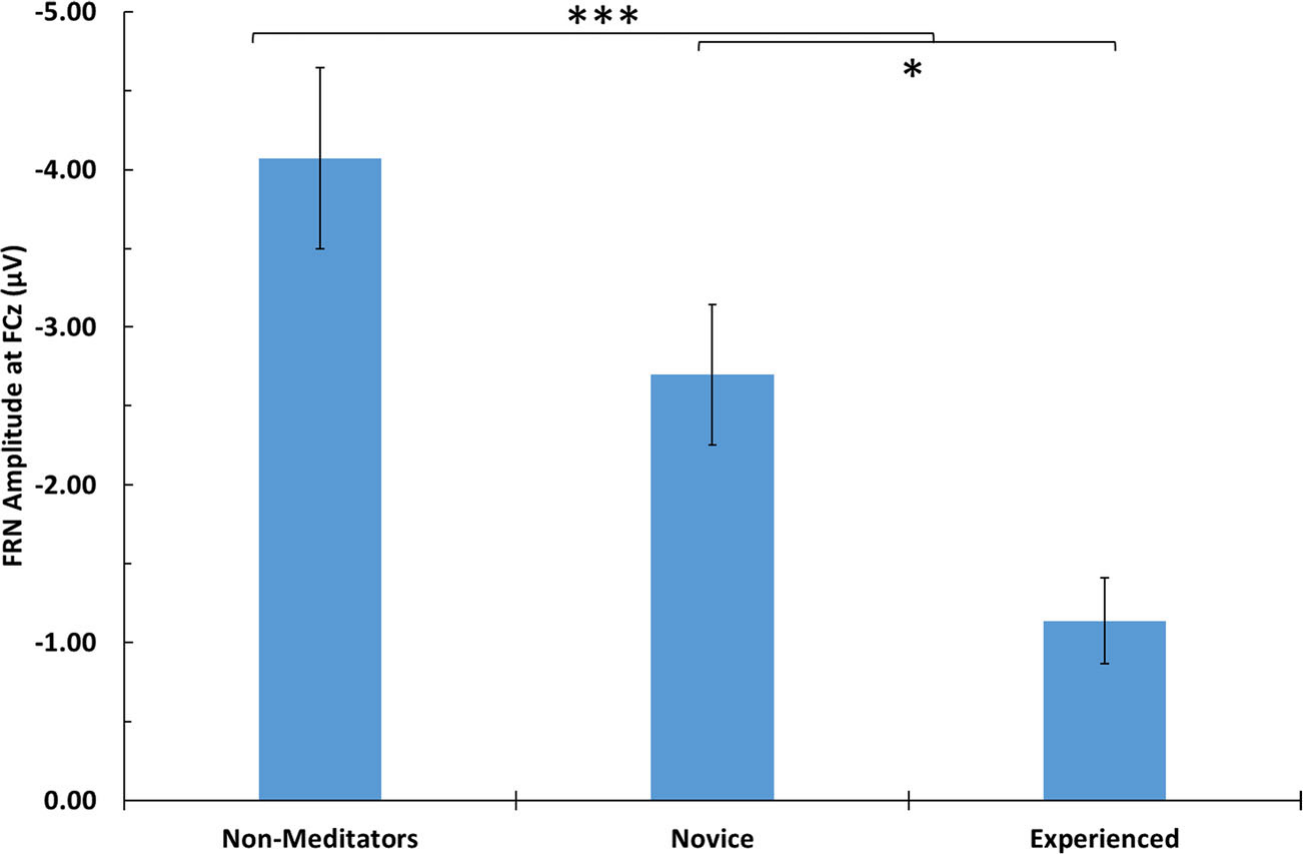
FRN amplitude at FCz as a function of FAM experience; error bars are *SEM*. All three groups differed significantly from one another, indicating that FRN amplitude decreases with meditation experience. *p ≤ .05. **p ≤ .01. ***p ≤ .001

The nonmeditation group had the highest amplitude FRN (*M* = −4.07 μV, *SE* = 0.57 μV; see Fig. 2a), peaking at 279 ms poststimulus, followed by the novice group (*M* = −2.70 μV, *SE* = 0.44 μV; see Fig. 2b), peaking at 272 ms poststimulus, and finally the experienced group (*M* = −1.14 μV, *SE* = 0.27 μV; see Fig. 2c), peaking at 263 ms poststimulus. The FRN was significantly different from zero for all groups: nonmeditators, *t*(11) = −7.09, *p* < .001; novice meditators, *t*(11) = −6.09, *p* < .001; experienced meditators, *t*(10) = −4.19, *p* = .002. An independent ANOVA was performed to examine the effect of meditation experience on FRN amplitude. As expected, analysis of these data revealed that there was a main effect of meditation experience on FRN amplitude, *F*(2, 32) = 10.18, *p* < .001, ƞ_p_^2^ = .39 (see Fig. 3). The timing and frontocentral scalp distribution (see Figs. 4 and 5) of these ERPs is characteristic of the classic FRN (Baker & Holroyd, 2011; Walsh & Anderson, 2012). There was no effect of meditation experience on FRN latency, *F*(32) < 1.

Planned contrasts revealed that there was a significant difference between nonmeditators and meditators, *t*(32) = −3.88, *p* < .001, *r* = .57, and also between novice and experienced meditators, *t*(32) = −2.40, *p* = .022, *r* = .39. We added participant age to the model to determine if this had an effect on the FRN. The ANOVA revealed that while there was still a significant effect of meditation experience on FRN amplitude, *F*(2, 31) = 9.87, *p* < .001, ƞ_p_^2^ = .39, there was no significant effect of age, *F*(1, 31) = 2.92, *p* = .10, ƞ_p_^2^ = .09. These results indicate that the FRN attenuates as meditation experience increases, and this effect is not explained by participant age. It is also worth noting that although weaker, when false feedback trials are included in our analysis, the direction of our results remain unchanged.

**Fig. 6:**
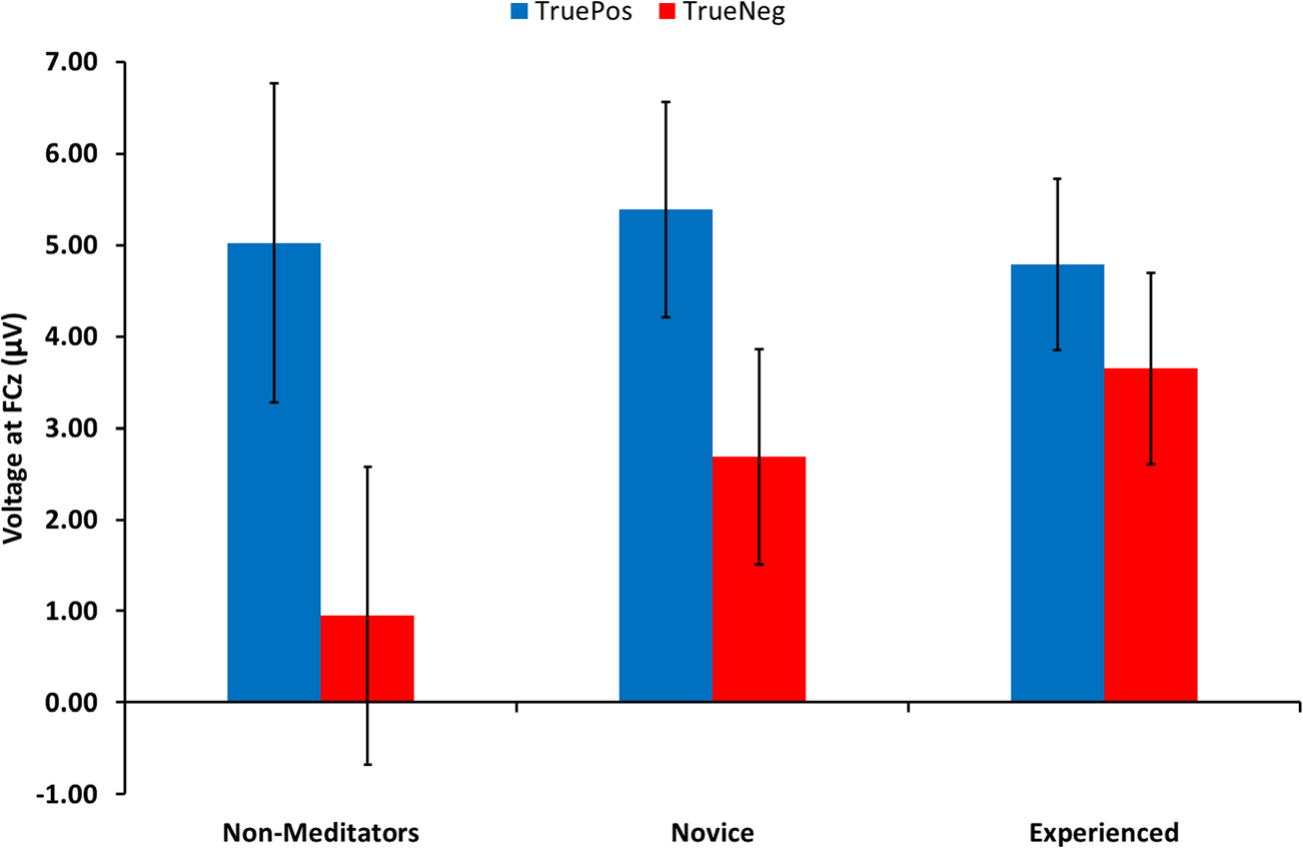
Condition-specific (true positive and negative feedback) elicited voltages, 50 ms either side of the difference wave peak at FCz, grouped by meditation experience. Error bars are *SEM*

As with the FLB data, we decomposed the difference wave by feedback type to determine whether the group differences in FRN were driven by an increase or decrease in either the positive-feedback-elicited or negative-feedback-elicited waveforms. The condition specific voltage at the FRN difference wave peak is summarized by feedback type for each group in Fig. 6 and Table 2. A 2 × 3 (Feedback Type × Meditation Group) mixed-design ANOVA was conducted and revealed a main effect of feedback type, *F*(1, 32) = 84.04, *p* < .001, ƞ_p_^2^ = 0.72, confirming that negative feedback elicited a more negative-going waveform than positive feedback did in all groups. While there was no main effect of meditation experience on FRN voltage, *F*(2, 32) = 0.44, *p* = .65, there was a significant feedback type by meditation group interaction, *F*(2, 32) = 10.18, *p* < .001, ƞ_p_^2^ = .39, indicating that meditation experience differently affects positive-feedback-elicited and negative-feedback-elicited voltage. Post hoc independent ANOVA was performed on the condition specific ERPs to attempt to explicate the significant interaction. Neither the positive feedback ERP, *F*(2, 32) = 0.05, *p* = .95, ƞ_p_^2^ < .01, nor the negative feedback ERP, *F*(2, 32) = 1.07, *p* = .36, ƞ_p_^2^ = .06, revealed a significant effect of meditation experience on ERP voltage. Thus, despite a possible trend-level effect of meditation experience on negative feedback voltage, our sample lacks the power to explain the interaction further.

**Table 2:** Condition-specific ERP voltages

Condition	Group	Voltage (μV)	*SE*
Negative feedback	Nonmeditators	0.95	1.63
	Novice meditators	2.68	1.17
	Experienced meditators	3.65	1.04
Positive feedback	Nonmeditators	5.02	1.75
	Novice meditators	5.38	1.17
	Experienced meditators	4.79	0.94

**Fig. 7:**
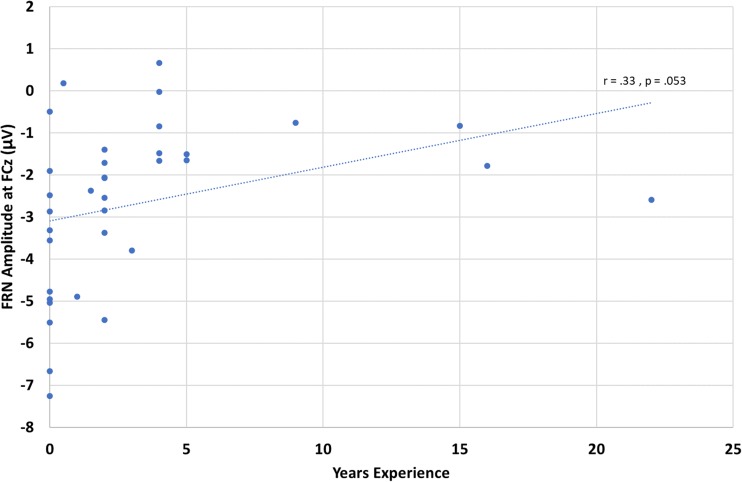
Linear regression models of the effect of meditation experience on FRN amplitude at FCz

As with the FLB, to investigate whether meditation experience predicts FRN amplitude, a linear regression was conducted (see Fig. 7 and Table 3). This analysis reveals a trend of reduced FRN amplitude as a function of increased meditation experience. Meditation experience did not significantly predict condition-specific ERP amplitudes, suggesting that this effect is driven by changes in both positive-feedback and negative-feedback processing.

**Table 3:** Summary of linear regression fit to condition-specific and difference wave FRN voltages at FCz at difference wave peak

Feedback type	*F*	*p*	*r*
Negative–positive	4.03	.053	.33
Positive	.08	.78	.05
Negative	.29	.60	.09

## Discussion

The aim of this study was to determine whether or not there was an association between FAM experience and behavioural and electrophysiological measures of reinforcement learning. As predicted, the behavioural data revealed that meditators were more positively biased feedback learners. These differences appear to be driven primarily by differences in negative rather than positive feedback processing. The ERP data revealed that the amplitude of the FRN was significantly smaller in meditators than in controls, and that these effects scale with meditation experience. Furthermore, these group differences cannot be explained by age, disease, or medication.

One potential explanation for these data is that striatal dopamine signalling varies as a function of meditation experience. First, PET has revealed that in experienced meditators, the act of meditating is associated with a significant increase in tonic dopamine levels in the striatum (Kjaer et al., 2002). Second, others using the probabilistic selection task (PST) reported a shift towards a positive FLB after using l-dopa to increase dopamine levels in Parkinson’s patients (Frank et al., 2004). Despite some controversy surrounding the reliability of the PST (Baker, Stockwell, & Holroyd, 2013) and its sensitivity to the pharmacological manipulation of dopamine levels in the brain in Parkinson’s patients (Grogan et al., 2017), there are a large number of studies demonstrating a trend of increased dopamine levels being associated with a more positive feedback-learning bias (FLB) and relatively lower dopamine levels being associated with more negative FLB (Cox et al., 2015; Frank & Hutchison, 2009; Frank & Kong, 2008; Frank & O’Reilly, 2006; Frank et al., 2004; Klein et al., 2007; Lighthall, Gorlick, Schoeke, Frank, & Mather, 2013; Smittenaar et al., 2012; Voon et al., 2010). Third, a recent PET study of healthy individuals has demonstrated that striatal D1 and D2 signalling respectively predicts learning from positive and negative outcomes on the PST (Cox et al., 2015). D1 receptors have low affinity and therefore respond more to phasic DA activity and less to tonic dopamine activity. D2 receptors, meanwhile, have high affinity and so are more strongly influenced by changes in tonic DA (Frank, 2005). Since phasic signalling is largely driven by D1 receptor activity which is correlated with Choose *A*, not Avoid *B*, performance on the PST (Cox et al., 2015), in the present study if differences in phasic signalling were solely or mostly responsible for the observed differences in feedback processing, we would expect to see a stronger correlation between meditation experience and variation in positive feedback processing than in negative feedback processing. However, the opposite appears to be the case: meditation experience seems to affect only negative-feedback learning. This suggests that the effect is driven by differences in striatal D2 signalling—not D1 signalling. Since D2 signalling is sensitive to changes in tonic dopamine or D2 receptor availability, our findings could indicate that one or both of these may increase as meditation experience increases. In other words, since tonic DA in the striatum increases during meditation and relatively higher tonic DA manifests as poorer Avoid *B* performance on the PST, our behavioural data could reflect persistent increases in striatal tonic DA or D2 receptor availability as a function of meditation experience.

This interpretation of our behavioural data is supported by our ERP data. There are a number of competing theories on FRN/ERN generation (San Martín, 2012; Walsh & Anderson, 2012); however, the highly influential reinforcement learning theory of the FRN links the FRN to the dopaminergic processing of feedback. The theory holds that errors and negative feedback result in dips in phasic dopaminergic activity, which disinhibits the anterior cingulate cortex (ACC) and generates the negative-going condition-specific component of the FRN, while positive feedback causes bursts of phasic dopaminergic activity, which inhibit the ACC and results in the more positive-going condition-specific component of the FRN (Holroyd & Coles, 2002; San Martín, 2012; Walsh & Anderson, 2012). Due to the tonic/phasic DA interaction in the basal ganglia (Frank, 2005), tonic dopamine levels affect this process. Higher levels bias toward the “go” pathway and inhibition of the ACC, thus attenuated FRN. Lower levels bias toward the “no-go” pathway and disinhibition of the ACC, leading to a larger FRN. For instance, Frank et al. (2005) found that, similar to our data, healthy participants who were positively biased (go) feedback learners on the PST also displayed an attenuated FRN compared with negatively biased (no-go) learners. Another study measuring the effect of a Gene × Feedback-Valence interaction on FRN generation during a gambling task reported a lower amplitude FRN in participants whose tonic DA levels were presumably higher due to having the MET/MET allele of the val158met COMT polymorphism (Marco-Pallarés et al., 2009). This pattern is also seen in a study which found that at baseline MET homozygotes had a lower amplitude FRN as compared with VAL carriers, and this pattern reversed when participants were given sulpiride, a D2 antagonist (Mueller et al., 2014). Taken together, these findings demonstrate that an attenuated FRN is observed in healthy individuals who have relatively higher tonic dopamine levels. In the present study, since FRN amplitude decreased as meditation experience increased, and at a trend level, at least, this appears to be driven mainly by differences in negative-feedback processing, our data may be indicative of meditators having higher tonic dopamine than do nonmeditators.

The possibility that meditation could elevate tonic dopamine over time is intriguing. Here, we would like to postulate a potential mechanism for future investigation: It may be that the continuous and sustained application of top-down attention to (and conflict monitoring of) internal and external sensory inputs during FAM practice results in the activation and maintenance of a feedback loop between the dlPFC, ACC, and basal ganglia. Activation of this feedback loop could account for the release of dopamine and activity in dopaminergic structures reported during various meditation practices (Hagerty et al., 2013; Kjaer et al., 2002). Over time, meditators’ repeated activation of this loop would result in Hebbian potentiation of the circuit, which could account for the increased density in the ACC (Fox et al., 2014; Tang et al., 2015) and the increased connectivity between the ACC and striatum that has been reported in recent structural imaging studies of FAM (Tang et al., 2010). The resulting increase in dopaminergic synapses would then result in higher tonic dopamine levels. These elevated tonic dopamine levels could partially account for the improved mood reported by meditators (Brown & Ryan, 2003; Davidson et al., 2003; Hagerty et al., 2013; Singleton et al., 2014). The effect of disrupting this dopaminergic corticolimbic-striatal circuitry has been demonstrated in a rat model where chronic exposure to dopamine receptor antagonists resulted in reductions in ACC volume over time (Vernon et al., 2014). From this view, our findings fit well with these previous studies and the notion of meditation potentiating an attention-activated dopaminergic corticolimbic-striatal feedback loop.

Despite the agreement of our data with the above outlined view, they are, however, in apparent contrast with the results of a study demonstrating that trait mindfulness did not predict the FRN (Teper & Inzlicht, 2014). It is important to note that their study correlated the FRN to trait mindfulness (as measured by the Philadelphia Mindfulness Scale; PMS) instead of FAM experience. Although practices with a FAM component have been shown to increase trait mindfulness, trait mindfulness alone does not necessarily imply any FAM experience—the PMS measures self-reported present-moment awareness and acceptance which may be influenced by FAM, but is also argued to be a naturally occurring trait (Cardaciotto, Herbert, Forman, Moitra, & Farrow, 2008). Using trait mindfulness to infer FAM experience would be a conflation of trait/state/practice, as mentioned in the introduction.

In a similar vein, a recent study reported evidence for lower striatal dopamine signalling in experienced meditators (Kruis, Slagter, Bachhuber, Davidson, & Lutz, 2016). They recorded the spontaneous eye-blink rate (sEBR) of 27 experienced meditators and 118 nonmeditators at rest and found that experienced meditators compared with nonmeditators had a significantly lower sEBR, which is associated with low striatal dopamine. At first glance this suggests that we should expect meditators to have low tonic dopamine, contrary to our view. However, in an earlier study Slagter, Georgopoulou, and Frank (2015) found that sEBR is inversely correlated with Avoid *B* performance on the PST, suggesting that low levels of dopamine are associated with high Avoid *B* performance, and, vice versa, high tonic dopamine is associated with poorer Avoid *B* learning, which is consistent with our findings. Crucially, however, sEBR in both studies is recorded at rest, not under the task-related cognitive load of the PST. Kruis et al. (2016) argue that low sEBR at rest in meditators may be indicative of increased stability (requiring lower levels of dopamine), while under cognitive load (i.e., task performance) this would imply more cognitive flexibility related to higher dopamine levels. Our data support this possibility; however, our paradigm did not provide the resting state needed for us to examine sEBR in our sample. Although further work is needed to investigate whether this rest/load hypothesis can explain the apparent discrepancy between our findings and those of Kruis et al., these studies provide evidence that striatal dopamine signalling may be altered in experienced meditators.

Despite the our data demonstrating a relationship between total amount of mediation practice and differences in reinforcement learning and feedback processing, further research is needed to determine whether these differences are caused by meditation. Given the literature demonstrating neuroplastic changes in the dopamine system with meditation practice, and that meditators need to practice to become proficient, we find the idea that meditation is responsible for these changes most credible. However, since we did not manipulate meditation experience in the current study, we cannot rule this out as an alternative explanation. Indeed, Kruis et al. (2016) failed to find an effect of an 8-week meditation intervention on sEBR in a sample of 36 naïve participants. They also failed to find a relationship between meditation experience in experienced meditators and sEBR. This could be seen as evidence contrary to our hypothesis that meditation causes differences in striatal dopamine signalling. It may be that preexisting differences in striatal dopamine lead meditators to self-select for meditation practice. However, this would not explain the relationship in the present study between total amount of practice time and Avoid *B* performance or FRN amplitude. It is possible that their intervention was not long enough, a point Kruis et al. raise, and that their experienced meditators had too much experience (minimum experience in Kruis et al. was 1,439 hours). For comparison, our sample ranged from 4 to 4,004 hours of practice, with a median of 260 hours. It may be that our data captures a critical cross-section in meditation-induced plasticity missed in Kruis et al.’s sample. Another possibility is that type of meditation may also play a role. Mindfulness-based stress reduction (Kabat-Zinn, 1990), used in the Kruis et al. study, combines both focused attention (FA) and open monitoring meditation (OM) styles (Lutz et al., 2008), as well as yoga. The emphasis on single pointed attention in FA meditation (e.g., samatha, jhana meditation practices) may be more strongly associated with classically dopaminergic phenomenological effects of intense pleasure (e.g., Hagerty et al., 2013).

Finally, despite a growing body of evidence implicating alterations in dopaminergic activity in meditators, it is possible that our data could be explained by differences in other neurotransmitter systems. For example, while dopamine is believed to play a key role in FRN generation, increased levels of norepinephrine have been shown to amplify the ERN while serotonin may also have a modulatory effect (Jocham & Ullsperger, 2009). Serotonin has also been shown to influence the processing of reward value (Seymour, Daw, Roiser, Dayan, & Dolan, 2012).

Thus there is a clear need to address these questions in future work which manipulates both striatal dopamine and (preferably focused attention) meditation experience in a medium to long-term randomized controlled intervention. Determining whether meditation practice elevates tonic dopamine levels is crucial to our understanding of a widespread behaviour, how it may interact with disease and drugs, and presents the possibility of potentially low-cost behavioural interventions which could be used as an adjunct to pharmacological treatment of certain dopaminergic disorders.

In closing, the present study is, to the best of our knowledge, the first study to demonstrate that reinforcement learning and FRN amplitude vary as a function of total meditation experience. To explain these findings in the context of the literature, we posit a theory that meditation causes increases in tonic dopamine levels (or D2 receptor availability) in the striatum. We then consider alternate explanations, and thus reveal the need for further study to determine whether we might increase our tonic dopamine levels by learning to pay better attention.

## Appendix: Reported effects are robust to alternate quantifications of FAM experience

Since retrospective self-reported mediation experience can vary in intensity that is not entirely captured by the number of years of practice alone, we investigated the effect of two other quantifications of meditation experience on our results. The first was to multiply participant’s years of experience with their self-reported weekly meditation frequency and duration to get an estimate of their total hours of experience. Our third method calculates meditation experience, in hours, in the last 6 months, assuming self-report frequency and duration was constant. In all cases, a median split on meditation experience was used to separate participants into the novice and experienced meditators categories. The results of these different approaches can be seen in Table 4. Ultimately there is no perfect way to retrospectively assess the amount or quality of meditation experience, and this is a limitation of this type of study. However, it is worth noting that for both measures total years of experience accounted for the most variance in the data, and also that the effects reported were relatively robust to how meditation experience was quantified.

**Table 4:** Summary of the effect of different quantifications of meditation experience

Grouping variable	*N*			Bias			FRN amplitude		
	Control	Novice	Experienced	*F*	*p*	ƞ^2^	*F*	*p*	ƞ^2^
Total years	12	12	11	3.50	.042	0.18	10.18	<.001	0.39
Total hours	12	12	11	3.14	.06	0.16	8.05	.001	0.34
Last 6 Months	12	11	12	3.14	.06	0.16	7.45	.002	0.32

## Footnotes

1 Our findings are relatively robust to alternative quantifications of FAM experience. See the Appendix for details and discussion.
